# Substrate and Moisture Affect the Pupation Depth of the Corn Silk Flies *Chaetopsis massyla* and *Euxesta eluta* (Diptera: Ulidiidae)

**DOI:** 10.3390/insects14110838

**Published:** 2023-10-28

**Authors:** Sandra A. Allan

**Affiliations:** United States Department of Agriculture, Agricultural Research Service, Center for Medical, Agricultural and Veterinary Entomology, 1700 SW 23rd Drive, Gainesville, FL 32608, USA; sandy.allan@usda.gov

**Keywords:** soil type, moisture, pupation, larvae, behavior

## Abstract

**Simple Summary:**

Corn silk flies are among the major pests of fresh market sweet corn production in tropical and semi-tropical regions. They have been reported as particularly impactful in Argentina and southern and central Florida. Insecticides are used heavily to protect crops; however, lapses in treatment or pest resistance can result in sufficient damage to render the cobs unmarketable. To provide better insight into the development of alternative management strategies, research was conducted to better understand the pupation behavior of two major species of corn silk flies. In a laboratory study, three soil types were tested across six different levels of moisture to evaluate the pupation behavior of the flies. Both soil and moisture levels impacted pupation depths, and these factors are important for optimizing management strategies such as tilling or the use of parasitoids as biological control agents.

**Abstract:**

Corn silk flies, or picture-winged flies (Diptera: Ulidiidae), are important pests of fresh market sweet corn in commercial production areas in southern Florida. Issues with pest management related to insecticide resistance, problems in insecticide application, and alternate crop population sources constitute a significant challenge for the protection of developing corn ears. Developed larvae leave cobs and pupate in the soil; however, relatively little is known about these behaviors. In this study, two soil types collected from fields were compared with sand under six different moisture levels in the laboratory to determine the pupation depths of the larvae. Comparisons were carried out concerning the pupation depth of *Chaetopsis massyla* and *Euxesta eluta*, which are major pest species in Florida. Both soil type (muck, loamy sand, and sand) and moisture levels (0, 10, 25, 50, 75, 100% field holding capacity) significantly affected pupation depth, with shallow pupation observed under dry or saturated wet conditions. The addition of structures such as pipe cleaners simulating corn roots resulted in deeper pupation under most conditions.

## 1. Introduction

In regions in Florida where fresh market sweet corn is produced, corn silk flies (Diptera: Ulidiidae) are considered severe pests, rendering cobs unmarketable and resulting in annual losses of millions of dollars [1]. The primary pest species are the tropical and semi-tropical species *Euxesta eluta* Loew, *Euxesta stigmatias* Loew, and *Chaetopsis massyla* Walker [2]. While the primary economic impact is on sweet corn, other plants that support fly populations include crops (green peppers, tomato, and sugar cane), as well as several species of weeds [3]. In corn, flies preferentially oviposit on corn silk with larvae migrating and feeding on developing corn kernels [4]. Upon completing development, larvae migrate with most leaving the cob and pupating in the soil [5,6].

Fly control for reducing damage to levels that do not negatively impact harvested cobs is challenging. Extensive scouting is required to guide the application of broad-spectrum insecticides [7]. Recently, resistance to common pyrethroids has been reported [8,9]. Further challenges in control may be related to inconsistency in the performance of insecticides [9], application error, poor timing [10], staggered planting [11], and the maintenance of untreated non-crop areas of refuge [3]. Control efforts are primarily targeted against the ovipositing adults as larvae are difficult to treat within a developing cob. Given these problems, there is an urgent need to develop an integrated pest management system that targets the pupae in the soil [11]. A better understanding of the pupation behavior of flies can serve as the basis for the development of control strategies involving parasitoids, predators, or soil disruption.

Relatively little is known about the pupation behavior of corn silk flies. The mean pupation depth of *E. euxesta* in the laboratory was reported as 2.5 cm [5], and a subsequent field study revealed that post-harvest crop destruction through mowing and disking could reduce some adult fly emergence, although results were inconsistent [6]. The pupation depth of flies has been reported to vary with types of soil [12,13], moisture levels [12,14,15,16,17], soil compaction [17], shade [18], and temperature [12], although the impact of these factors may differ between species. A better understanding of the factors affecting pupation behavior in corn silk flies would be useful for the consideration of the use of pupal parasitoids, predators, or methods of crop destruction that could be incorporated into an IPM program. In this study, the impact of different soils, moisture levels, and the presence of physical structures were evaluated against pupating *Chaetopsis massyla* and *Euxesta eluta*.

## 2. Materials and Methods

### 2.1. Insect Rearing

Two species of corn silk flies, *Chaetopsis massyla* and *Euxesta eluta*, known to be significant pests of sweet corn in Florida [2], were laboratory-reared for these tests. Flies were maintained on 10% sugar water and provided with a corn/agar diet [6] with green pepper sections [19]. For assays, late instars that crawled or jumped out of cups prior to pupation [20,21] were collected. Larvae for assays were collected from paper towels or diet cups in rearing containers [19]. Colonies were held in environmental chambers at 27 °C, 60–80% RH, and a 16:8 L:D photoperiod.

### 2.2. Substrates and Moisture Levels

The substrates used in this study represent two soil types in which both corn silk fly species are abundant in corn fields at the University of Florida, Institute of Food and Agricultural Sciences, Everglades Research and Education Center (Belle Glade, Palm Beach County) and the University of Florida Institute of Food and Agricultural Sciences, Plant Science Research and Education Center (Citra, Alachua County). Soil samples were collected from at least two locations in corn fields at each site. The samples comprised the top 30 cm of soil. Soil from Belle Glade was muck soil (histosol) and that from Citra was loamy sand, and their compositions are presented in Table 1. The muck soil (sapric histosol) consists of highly decomposed organic matter derived from the drainage of wetlands or flooding in the Everglades agricultural area [22]. For comparison, commercial sand (Quikrete Play Sand, Quikrete Companies, Atlanta, GA, USA) was also included.

For field-collected samples, plant roots and other types of material were removed, and soil was sieved through a brass sieve (80 mesh, 0.18 mm). The substrates were air-dried then oven-dried at 55 °C for 48 h and held in sealed containers until use. Field holding capacity (FHC) for water for each soil type was determined following Jenkinson and Paulson [23], which involved wetting dry soil until saturation and quantifying the amount of water added [24] required to saturate soil to the point of run-off. Once the field loading levels were determined, the substrates were prepared 24 hours before use at 10%, 25%, 50% 75%, and 100% (*w*/*w*) field holding capacity and placed in sealed containers to ensure full wetting. Substrates were mixed evenly before use, and those with no water added were considered 0% FHC. Soil moisture levels were determined using a moisture analyzer (A&D Company, Ltd., San Jose, CA, USA, model MX-50).

### 2.3. Bioassays

**Figure 1:**
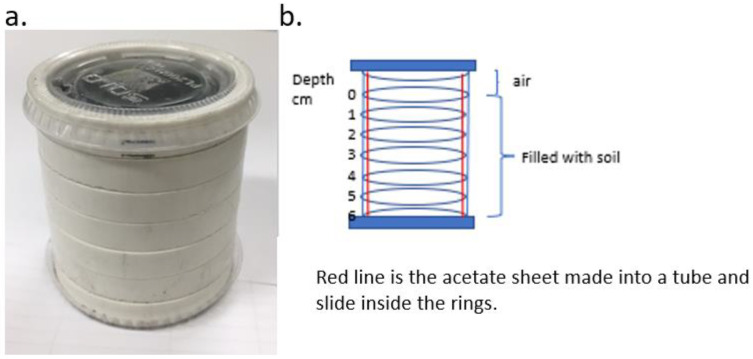
Stacked PVC ring assay setup used for experiments: (**a**) picture of intact ring stack and (**b**) diagrammatic representation of the ring stack.

A stacked ring system [14,24] was developed and consisted of 7 stacked rings made of PVC tubing (5.25 mm ID, 6 mm outer diam and 1 cm thick) (Figure 1a). The bottom ring was placed in a plastic lid (Solo PL2, Lake Forest, IL, USA) that fit snugly, and 6 rings were stacked on top. A liner made of an acetate sheet (20 cm × 6.5 mm tall) was formed into a cylinder and placed inside the rings and served to hold and stabilize the ring stack (Figure 1b). A substrate was added to each ring to the top of the 6th ring, and a lid was added to the top of the 7th ring, which provided 1 cm of air space. All substrates were used at similarly low levels of compaction.

### 2.4. Effect of Soil Type and Soil Moisture on Pupation Depth of Flies

The stacked ring system was used to evaluate the effect of different soils and moisture levels on the pupation depth of the two fly species. Late third instars of these species actively migrate out of their rearing material [5]. For these assays, larvae were collected as they migrated out of the rearing media and placed on top of the substrate and the lid was positioned to contain the larvae. After 7 days, the lid was removed, and the acetate liner very slowly and carefully removed. The stack of rings was placed in a pan, and sequentially starting with the top ring, each ring was slid to one side, and the substrate was scattered and viewed for pupae, which were counted. Pupal numbers were obtained for each ring depth. For these tests, the three types of soil were tested at 0, 10, 25, 50, 75, and 100% FHC. The acetate sheet and plastic lids served to retain moisture levels during the test. Tests were conducted in environmental chambers at 25 °C, 70–80% RH, and under a 16:8 L:D photoperiod. Ten larvae were placed on each ring stack, and two groups of six ring stacks were tested for a total of twelve replicates.

### 2.5. Effect of Physical Structure

To mimic the effect of corn roots and other structures in soil, two sections of chenille pipe cleaner (Horizon Group, Shanghai, China) (0.5 mm diam, 7 cm long) were inserted vertically 1 cm apart in the center of ring stacks containing different soil types (muck, loamy sand, and sand) at 0 and 25% FHC. Each ring stack received 10 larvae, and two groups of ring stacks were tested for a total of 12 replicates. At the end of the test, the pipe cleaners were slowly pulled out of the soil so as to not dislodge pupae. The number of pupae at each depth was determined as described above.

### 2.6. Statistical Analysis

The mean depth of pupation of individual pupa was calculated for each ring stack and used to examine the effects of different soil and moisture conditions for each species. Fixed-effect factorial design two-way analysis was conducted for each species with soil, moisture, and soil*moisture interactions using PROC GLM, and the residuals were plotted to assess the model’s fit (SAS Institute, Cary, NC, USA). The factors considered were soil and moisture, and the depth of pupae was the response variable. If significant differences were discovered within variables, mean comparisons were performed (Tukey’s range test). For comparisons in pupation depths between the presence and absence of pipe cleaners, paired *t*-tests were conducted. Prior to testing, data were tested for normality (Shapiro–Wilk, *p* > 0.05).

## 3. Results

**Figure 2:**
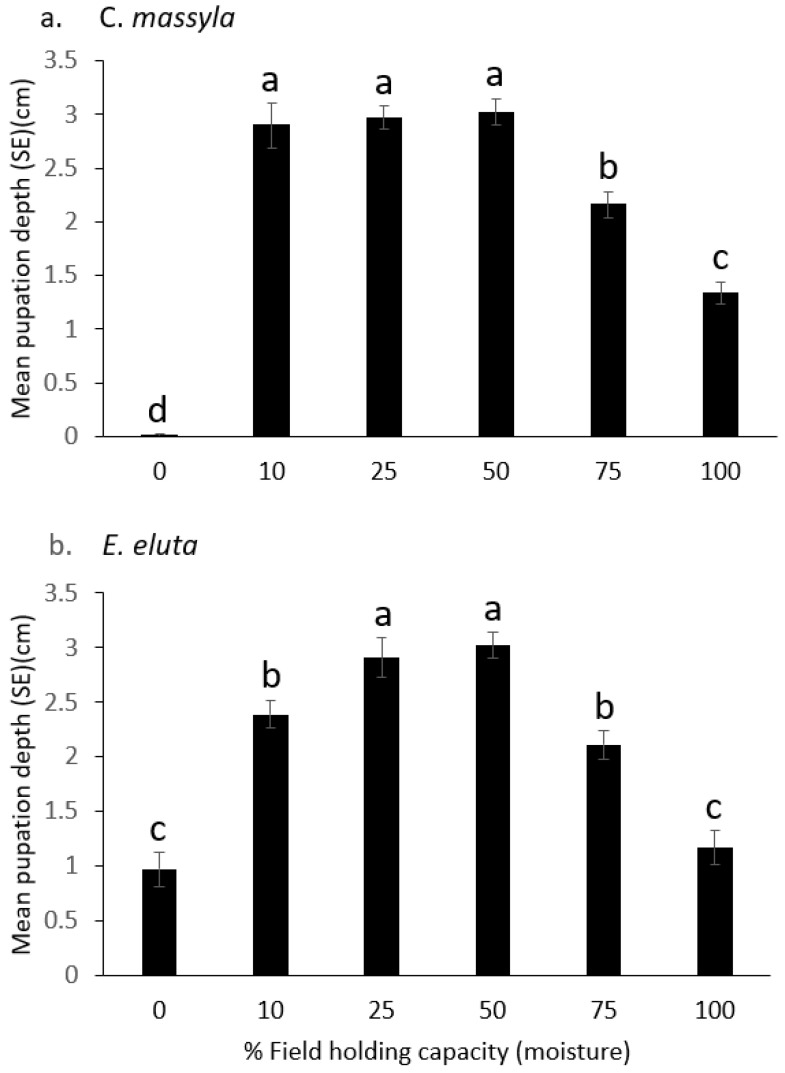
Comparison of the depth of pupation (cm below surface) at six levels of field holding capacity (moisture) of (**a**) *Chaetopsis massyla* and (**b**) *Euxesta eluta*. 0 depth indicates soil surface. Within each species, means with different letters are significantly different (*p* < 0.05).

For both *Chaetopsis massyla* and *Euxesta eluta*, there were significant main model effects (Table 2) with both soil and moisture as significant factors. Additionally, there were significant soil*moisture interactions. Across all moisture levels, there were significant differences in pupation depth between soils for both *C. massyla* (F = 4.10, df = 2215, *p* < 0.01) and *E. eluta* (F = 6.85, df = 2215, *p* < 0.01). Pupation depths were greater in muck soil than in loamy sand and sand. Across all soil types, there also were significant differences in pupation depth for *C. massyla* (F = 33.84, df = 5215, *p* < 0.0001) as well as for *E. eluta* (F = 91.46, df = 5215, *p* < 0.0001). The deepest pupation for *C. massyla* was at 10, 25, and 50% FHC, followed by 75% FHC, 100% FHC, and then 0% FHC (Figure 2). For *E. eluta*, the deepest pupation was at 25% and 50% FHC, followed by 10% and 75%, and then 0% and 100% FHC (Figure 2).

**Figure 3:**
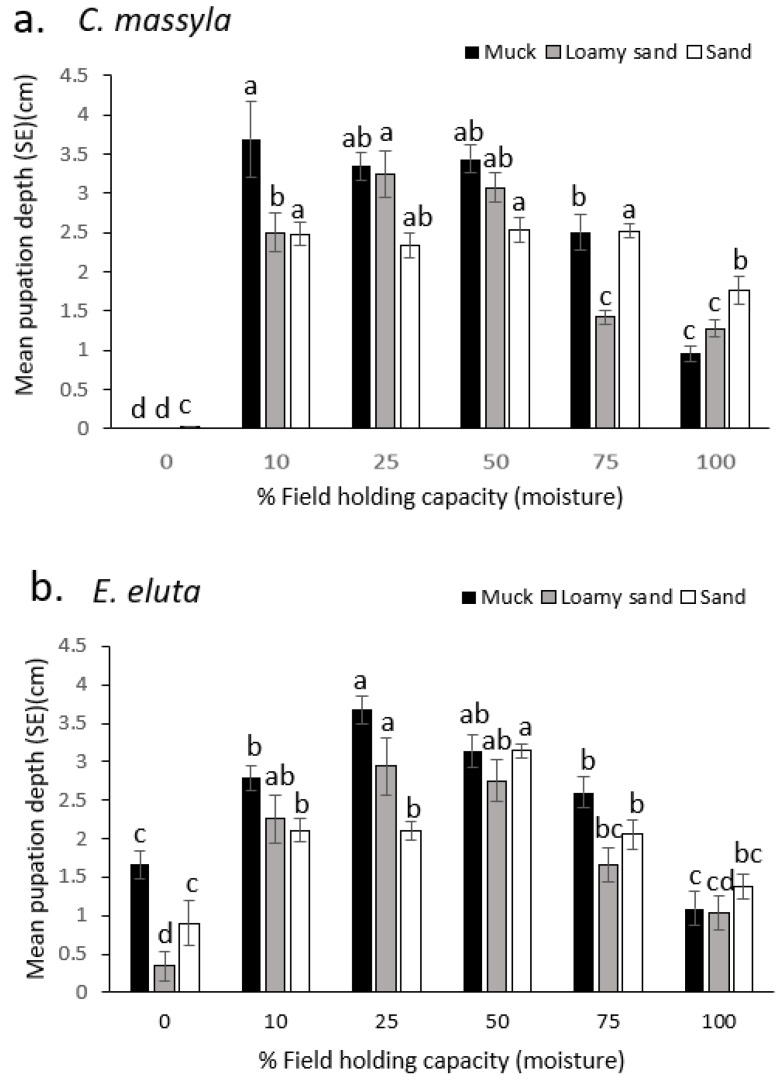
Comparison of the depth of pupation (cm below surface) of (**a**) *Chaetopsis massyla* and (**b**) *Euxesta eluta* in three soils at six levels of field holding capacity (moisture), with 0 depth indicating the soil surface. Within each soil type, means with different letters are significantly different (*p* < 0.05).

Overall, for both species, pupation was deeper in the soil when moisture (% FHC) was lower (10–50%), and then was closer to the surface at the highest moisture levels tested (Figure 3). In dry soil (muck, loamy sand, and sand), *C. massyla* pupated at the surface (Figure 3a). An increase in moisture up to 10% FHC dramatically increased the pupation depth in all soils. For muck soil, the greatest depths were obtained with 10, 25, and 50% FHC, with pupation occurring closer to the surface for 75% and 100% FHC. For loamy sand, the greatest depth of pupation was at 25% and 50% FHC, followed by 10% FHC. Pupation was closer to the surface with 75% and 100% FHC (Figure 3a). In sand, pupation was deepest and similar between 10, 25, 50, and 75% FHC, with more shallow pupation at 100% FHC and mostly at the surface at 0% FHC.

For *E. eluta*, in dry soil of any type, pupation generally occurred within the top 1.5 cm (Figure 3b). In muck soil, the deepest pupation occurred at 25% and 50% FHC, with more shallow pupation at 10% and 75% FHC and the shallowest pupation at 100% and 0% FHC. In loamy sand soil, the deepest pupation occurred at 10, 25, and 50% FHC, with more shallow pupation at 75%, 100%, and 0% FHC. In sand, the deepest pupation was at 50% FHC, with more shallow and similar pupation depths at 10, 25, 75, and 100% FHC. Pupation with 100% and 0% (dry) FHC sand was similar and generally occurred at 1–1.5 cm below the surface (Figure 3b).

**Figure 4:**
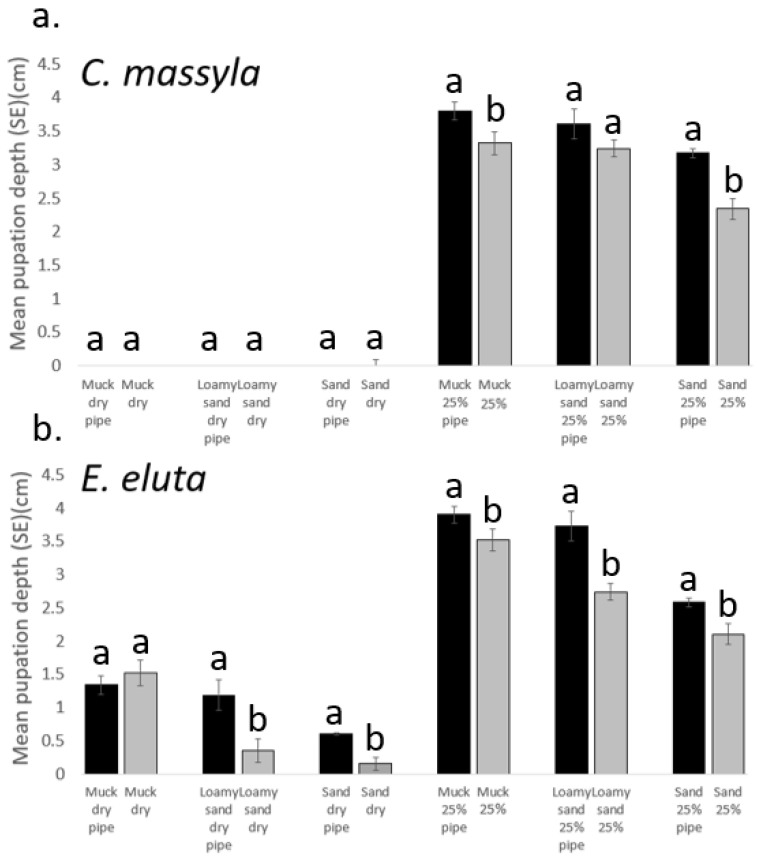
Comparison of the effect of pipe cleaners (pipe) on depth of pupae (cm below surface) of (**a**) *Chaetopsis massyla* and (**b**) *Euxesta eluta* in three soils at 0% (dry) and 25% field holding capacity (moisture), with 0 depth indicating soil surface. Within each paired comparison, with and without pipe cleaners, means with different letters differ significantly (paired *t*-test, *p* < 0.05).

The addition of pipe cleaners as surrogates for corn roots generally resulted in pupation at deeper levels for both species (Figure 4). For *C. massyla*, under dry soil conditions (0% FHC), there were no effects with the presence of the pipe cleaners as pupation occurred almost entirely at the surface. Under 25% FHC, treatments with pipe cleaners resulted in deeper pupation by *C. massyla* with muck and sand but not for loamy sand soil. In contrast, pupation by *E. eluta* occurred deeper under all moisture and soil conditions except for dry muck soil (Figure 4).

## 4. Discussion

A better understanding of the depth of pupation in the soil is important as a basis for developing potential management strategies. When infesting corn, corn silk flies have been reported to develop in the cobs with some pupation within the cob, but most pupate outside of the cob and in the surrounding soil [5]. Clearly, this is a function of a complex number of factors; however, soil type and moisture have a significant impact on pupation by *Euxesta eluta* and *Chaetopsis massyla*.

When pupating in soil, cyclorrhaphous Diptera generally pupate within the top several centimeters with occasional pupation at 5–6 cm [12,13,14,17,18]. In this study, the pupation of *C. massyla* ranged from 0 cm (surface) under dry conditions for all soils to a maximum mean depth of 3.5 cm for 10% FHC in muck soil. For *E. eluta*, all pupation occurred under the soil surface, with the shallowest for loamy sand at 0.25 cm and the deepest for muck soil at 25% FHC. The only prior report of the pupation depth of Ulidiidae indicated that *Euxesta eluta* pupates at a mean depth of 2.5 cm in Dania muck soil at an unspecified moisture level [5]. The results of the present study are very similar with depths of 2.5 cm for 10% and 75% FHC, both of which were similar to 50% FHC. For tephritid flies, the pupation depth is generally reported within 3 cm of the surface [12,13,14,17,18,25], with occasional depths of 4 cm or 5 cm reported [16,17]. Most pupation by *Drosophila suzukii* (Matsumura) occurs just below the surface (down to 2.5 cm), with none occurring on the soil surface [26].

Soil moisture had a significant impact on the depth of pupation for both species of corn silk fly in the current study. Pupation occurred at the shallowest depths and on the surface for *C. massyla* when soils were dry, and with increasing moisture, pupation was deeper. As moisture levels neared saturation (100% FHC), pupation occurred closer to the surface. Overall, the trend of more pupation at or near the surface in dry soil and at increased depths with increased moisture has been reported for several tephritids [12,14,16,17,27] as well as *D. suzukii* [26]. The effect of increased moisture on pupation also may vary between soil types [12].

Extremes of soil moisture can impact pupal survival [16,23], but this varies between species [23,28]. Both may also influence the selection of pupation depths. High-moisture soils may have a shortage of oxygen and thus be hazardous for survival [29]. The burrowing rate of the lepidopteran, *Spodoptera frugiperda* (J.E. Smith), decreased significantly at an 80% moisture level [29]. Reduced pupation at high moisture levels has also been reported for the swede midge (a cecidiomyiid) [30]. Renkema et al. [31] speculated that under saturated conditions, larvae are aerobically restricted from burrowing deeper, resulting in pupation on or near the surface. When soil moisture reaches 100%, the reduced availability of oxygen is considered to be a factor leading to surface pupation [12]. In a field study in muck soil in southern Florida, reduced emergence of corn silk flies was reported in fields that received heavy rains and were waterlogged [6]. The efficacy of using tillage to reduce emerging flies through disturbance and relocation of pupae was seasonally inconsistent possibly due to seasonal factors such as temperature and rainfall [6].

The production of fresh market sweet corn in Florida occurs in several areas with large-scale production in muck soils and smaller-scale production in soils such as sandy loam. In some prior studies, soil type did not impact pupation depth [17,29]; however, for corn silk flies in this study, the pupation depth was consistently deeper in muck soil than in loamy sand or sand soils, with similar results between *C. massyla* and *E. eluta*. The finer particle size of the muck soil may have facilitated deeper burrowing by larvae. Soil type can greatly influence the pupation depth of the tephritid *Rhagoletis mendax* Curran with deeper pupation in moist sand compared with moist soil; however, there were no differences between dry substrates [31]. For the olive fruit fly *Bactrocera oleae* (Gmel.), pupation occurred at lower depths in sandy loam of limestone origin compared with other sandy loam soils [12], possibly due to the finer texture of the limestone soil, whereas the other loam soils had a higher sand content. For *D. suzukii*, in a comparison of different soil types at 40% water holding capacity, there was a significant impact of soil type on pupation depth, with nearly all (96%) pupae in the upper soil layer of sandy soils (0–0.6 cm) compared with the lower layer (0.7–1.2 cm). In the upper layer of loam and clay soils, 78% and 58% of pupae were present, respectively [32].

In this study, the presence of physical vertical structures (pipe cleaners) as surrogates for corn roots generally resulted in deeper pupation. Larvae are highly thigmotactic and seek crevices prior to pupation (SAA, personal observation), and the presence of vertical structure in the soil presumably facilitated access to lower depths for pupation. Under dry conditions, the use of physical structure had no effect on pupation depth by *C. massyla* for any of the soils tested, indicating that moisture was a stronger factor than soil type in regulating pupation behavior. In contrast, for *E. eluta*, the addition of structure resulted in significantly deeper pupation for the soils with high sand content (sand and loamy sand) compared with the muck soil, presumably because it enhanced the passage of larvae to lower depths. When soil moisture was increased to 25% FHC, pupation depths were deeper in the presence of 25% FHC for all soils and both fly species. The influence of physical vertical structures such as roots may enhance the depth of pupation achieved by the fly pupae and may potentially impact the access of parasitoids or predators to pupae as well.

Pupation depth is affected by multiple factors, including moisture and soil type. In turn, the depth of pupation can influence the survival and ultimate emergence of adult flies through a variety of factors. Parasitoid wasps can serve as important biological control agents of cyclorrhaphous flies, and this is particularly noteworthy for pupal parasitoids for muscoid flies [33,34]. Some species of pupal parasitoids of house flies appear to be of limited utility for control of pupae that are buried, while others have been reported to parasitize pupae under the soil surface [32,35,36]. Recently, several pupal parasitoid species evaluated against *E. eluta* and *C. massyla* were demonstrated to be effective parasitoids [19], and future studies on the impact of soil type and moisture on the efficacy of parasitoids may provide a beneficial tool for management strategies for corn silk flies. Pupal depth also impacts the efficacy of parasitic nematodes [18] as well as predators [25,37,38].

Most large-scale commercial production of fresh market sweet corn in Florida occurs in areas with predominantly muck soils and in the presence of irrigation or fresh rainfall. Under these conditions, during times of crop production, the soil would remain in a range from moderately moist to saturation. These conditions for *E. eluta*, one of the most predominant species in the Florida fresh market sweet corn production areas, would result in pupation depths ranging from 1 to 3.5 cm. Even under dry conditions, pupation occurs under the soil surface. These depths may afford the protection of pupae from parasitoids or predators that are effective mostly at the soil surface. For *C. massyla* under most conditions, pupae are found under the soil surface. However, under dry conditions, this species pupates at the surface leaving pupae more prone to mortality factors. A better understanding of pupation depth can assist in the assessment of potential effects of parasitism and predation, the development of pupal surveillance methods, and potential soil intervention approaches.

## Figures and Tables

**Table 1:** Characteristics of soil substrates used in experiments.

Substrate	Particle (%)				Field Holding Capacity (% Moisture)	Moisture Content (%) at Each Field Holding Capacity Level					
	Sand	Silt	Clay	Muck		0%	10%	25%	50%	75%	100%
Pahokee Association histosol (Dania muck) (Palm Beach Co.)	0	0	0	100	119.17	0	21.2	39.7	49.4	56.3	61.7
Arredondo series ultisol (loamy sand) (Alachua Co.)	93	4	2.7	0	42.08	0	2.7	4.1	17.9	23.3	32.5
Sand (commercial)	100	0	0	0	27.82	0	0.6	2.2	11.9	17.1	61.7

**Table 2:** Results of analysis of variance for effects of soil type (muck, loamy sand, and sand) and moisture (0, 10, 25, 50, 75, and 100% field holding capacity) on mean pupation depth of two species of corn silk flies.

Species	Source	DF	F-Value	*p*
*Chaetopsis massyla*	Main model	17	15.20	<0.0001
	Soil	2	8.87	0.0002
	Moisture	5	125.45	<0.0001
	Soil*moisture	10	7.23	<0.0001
	Error	198		
*Euxesta eluta*	Main model	17	27.40	<0.0001
	Soil	2	13.79	<0.0001
	Moisture	5	40.73	<0.0001
	Soil*moisture	10	2.72	<0.0037
	Error	198		

## Data Availability

Data are available upon reasonable request.
